# Semi-Supervised Autoencoder for Chemical Gas Classification with FTIR Spectrum

**DOI:** 10.3390/s24113601

**Published:** 2024-06-03

**Authors:** Hee-Deok Jang, Seokjoon Kwon, Hyunwoo Nam, Dong Eui Chang

**Affiliations:** 1 School of Electrical Engineering, Korea Advanced Institute of Science and Technology, Daejeon 34141, Republic of Korea; jhd6844@kaist.ac.kr (H.-D.J.); jun115533@kaist.ac.kr (S.K.); 2 Chem-Bio Technology Center, Advanced Defense Science and Technology Research Institute, Agency for Defense Development, Daejeon 34186, Republic of Korea; hyunwoonam@add.re.kr

**Keywords:** chemical warfare agent, chemical gas classification, Fourier transform infrared, deep neural network, semi-supervised autoencoder

## Abstract

Chemical warfare agents pose a serious threat due to their extreme toxicity, necessitating swift the identification of chemical gases and individual responses to the identified threats. Fourier transform infrared (FTIR) spectroscopy offers a method for remote material analysis, particularly in detecting colorless and odorless chemical agents. In this paper, we propose a deep neural network utilizing a semi-supervised autoencoder (SSAE) for the classification of chemical gases based on FTIR spectra. In contrast to traditional methods, the SSAE concurrently trains an autoencoder and a classifier attached to a latent vector of the autoencoder, enhancing feature extraction for classification. The SSAE was evaluated on laboratory-collected FTIR spectra, demonstrating a superior classification performance compared to existing methods. The efficacy of the SSAE lies in its ability to generate denser cluster distributions in latent vectors, thereby enhancing gas classification. This study established a consistent experimental environment for hyperparameter optimization, offering valuable insights into the influence of latent vectors on classification performance.

## 1. Introduction

Chemical warfare agents represent highly toxic and perilous substances capable of inducing severe effects on the human body even in minute quantities. Many toxic chemical agents remain gaseous, colorless, and odorless at room temperature, posing challenges in promptly addressing chemical gas attacks or leaks. Hence, the early identification of and response to chemical gases are imperative tasks [1,2,3]. Additionally, the accurate classification of the specific chemical gas present in the atmosphere is crucial, as the approach to handling these gases varies with their types. Recognizing the significance of swift responses to chemical threats, researchers have focused on leveraging Fourier transform infrared (FTIR) spectroscopy [4,5,6,7,8,9,10,11,12]. This technique allows for the remote and non-contact analysis of materials. Through FTIR spectroscopy, the unique absorption or emission patterns in specific bands of the long-wavelength infrared region enable the identification of material types based on their molecular structures. Nevertheless, the observed spectrum is susceptible to background signals, light scattering, and noise inherent to equipment, making the clear identification and differentiation of chemical agents challenging. To address these challenges, recent studies have explored the application of machine learning and deep learning for FTIR sensing.

Yu et al. [13] employed an SVM to classify six classes, including five types of gases and a class that did not contain the other gases. They applied a preprocessing process, including converting the radiance spectrum to brightness temperature and noise removal [8] and baseline removal methods [8], achieving an enhanced performance by employing an SVM trained on preprocessed data. In a subsequent study [14], they added a process to remove the influence of background signals identified using principal component analysis (PCA) to the preprocessing method of the previous study and used this preprocessed data to train an SVM. The SVM trained with this preprocessed data achieved a higher performance than the correlation coefficients after removing background signatures [15], adaptive subspace detectors [16], and the SVM trained with preprocessed data in previous studies [13]. Nam et al. [17] classified gases using an SVM, discerning the presence or absence of classified gases based on SVM scores. Kim et al. [18] utilized deep neural networks and convolutional neural networks, outperforming the SVM as a comparative model. The ability of FTIR spectroscopy to identify unique spectral patterns based on material molecular structures extends beyond chemical gases to various materials. Ongoing research explores performance enhancement using machine learning and deep learning [19,20,21,22,23].

Given that measured spectra contain overlapping peaks not only from the specific band of the target material but also from background and surrounding materials, methods like feature selection and extraction have the potential to enhance the discrimination accuracy [24]. While methods using PCA have been studied [21], the use of deep learning autoencoders, expressing data characteristics as latent vectors, has gained traction. Jo et al. [24] classified agricultural products using an autoencoder and SVM, demonstrating an improved performance. The autoencoder was trained using the spectrum dataset, and the resulting encoder was employed as a feature extractor to train the SVM, exhibiting a superior performance compared to alternative feature extraction techniques like PCA or local linear embedding. Fine et al. [25] employed an autoencoder to utilize features with removed redundant information and noise. The features generated by the trained encoder were then employed in MLP training to classify functional groups. Chen et al. [26] proposed a model combining the encoder of a trained autoencoder and a classifier to classify orchid genotypes. Although many studies have employed autoencoders for extracting spectral features [27,28,29], the frameworks utilizing autoencoders in this manner have limitations, as the autoencoder and the model trained in a supervised manner are typically trained separately. While the autoencoder is trained to ensure that the reconstruction value produced through the encoder and decoder matches the input, the encoder obtained in this process may capture the semantics of the input data effectively, but this feature may not be optimal for supervised learning. To address these limitations, researchers have turned to semi-supervised autoencoders, combining both the autoencoder and supervised learning models for simultaneous training [30,31]. The semi-supervised autoencoder represents a unified framework that integrates an autoencoder with a task-specific supervised learning model attached to the latent vector of the autoencoder. Within this integrated framework, the autoencoder is trained to express not only the features for reconstruction from the input data but also the features for the attached supervised learning model, ultimately enhancing the performance of the attached task-specific model [32,33,34,35,36,37].

In this study, we propose a semi-supervised autoencoder for classifying chemical gases using FTIR spectra. The proposed model comprises a structure where a neural network-based classifier is attached to the latent vector, which is the encoder output of the autoencoder, training the classifier and autoencoder simultaneously. The classification performance of the proposed model was evaluated using nine types of FTIR spectra collected in a laboratory. A comparative analysis with existing machine learning methods, deep neural networks, and methods using an autoencoder as a feature extractor was conducted. The experimental conditions, including hyperparameter optimization and statistical performance analysis, were consistent across all models, providing insights into the impact of the latent vectors on the classification performance. Section 2 details the experimental data, the proposed model, and the hyperparameter optimization process. Section 3 presents the experimental setup and classification performance. Finally, Section 4 provides the conclusion.

## 2. Materials and Methods

### 2.1. Data Source

We employed FTIR spectral data collected from a chemical gas brightness temperature spectrum measurement system established at the Agency for Defense Development of the Republic of Korea. The measurement system comprises a custom-built remote chemical detection device (MSCAD, miniaturized standoff chemical agent detector) for capturing FTIR spectra, a gas chamber for collecting injected gas, and a black body used as a background. The MSCAD equipment has a field of view of 18 mrad, positioned at a distance of 3 m from the blackbody. The lens effective aperture is 15 mm. The chamber measures 2 m in length, with both the MSCAD equipment and the blackbody positioned at a distance of 0.5 m from the chamber. The process of collecting gas spectra was as follows: first, we ensured that the gas chamber window was within the field of view of the MSCAD measurement equipment. We aligned the MSCAD–chamber–blackbody setup so that the MSCAD ray passed through the gas chamber window and reached the black body. Subsequently, we set the temperature of the air inside the gas chamber and the temperature of the black body and injected chemical gas into the chamber. After introducing the gas, we waited for 20 s to allow mixing with the internal air and stabilization and then measured the brightness temperature spectrum of the gas at 35 Hz for approximately 20 to 40 s using the MSCAD device. The aforementioned process was conducted for nine types of chemical gases (cyclosarin (GF), lewisite (L), methanol, sarin (GB), nerve agent (VX), nitrogen mustard (HN3), soman (GD), sulfur mustard (HD), and tabun (GA)) with data collected at various concentrations for each gas by controlling the amount of substances injected into the chamber. For all gases except VX, the gas temperature inside the chamber was maintained at 40 °C, while the blackbody was set at 30 °C, resulting in a temperature difference of 10 °C between the blackbody background and the gas. In the case of VX, the gas temperature inside the chamber was adjusted to 50 °C, while the blackbody temperature remained at 40 °C, maintaining the same 10 °C temperature difference between the background and the gas. For security reasons related to the ongoing development of the spectrum measurement system, the names of the gases were pseudonymized as A–I.

To transform the collected spectrum into a dataset for training a neural network, we conducted a data preprocessing procedure and split the dataset into two subsets for training and testing datasets. We considered the spectrum range from the 739 ${\mathrm{cm}}^{-1}$ to 1260 ${\mathrm{cm}}^{-1}$ band, with a resolution of 1.6 ${\mathrm{cm}}^{-1}$ for the MSCAD measurement device. Therefore, the dimension $N_{i}$ of the input measurement spectrum $x\in R^{N_{i}}$ was 327. The spectrum *x* underwent a 16-frame moving average sequentially to remove noise. In the preprocessing process, min–max normalization and zero-mean normalization were applied sequentially to adjust the scale and bias of the dataset. Additionally, as the measured time varies for each experimental condition, 100 data points were randomly selected for each condition set in the experiment. The label $y\in R^{N_{C}}$ is a one-hot vector indicating the gas class to which the input *x* belongs, and $N_{C}$ was 9. Depending on the concentration in the dataset, high-concentration data were considered a training dataset, and low-concentration data were considered a test dataset. The higher the concentration of chemical gases, the higher the value in the unique peak band of the gas, allowing for the clear identification of the gas type in such cases. However, due to the potential fatal effects of chemical gases even in small amounts, accurate classification is necessary even in low-concentration gas situations. Therefore, we considered low-concentration data as the test dataset to evaluate applicability in a real environment. Table 1 shows the injected amounts and the number of data points for each gas included in the training dataset and test dataset.

### 2.2. Proposed Method

An autoencoder typically consists of an encoder that transforms input data into a low-dimensional latent vector and a decoder that reconstructs the input from the latent vector [38,39]. The autoencoder was trained in an unsupervised manner without labels. During the training step, latent vectors are expected to extract important features for reconstructing the same input data. This characteristic makes the autoencoder a representative model for representation learning. In various spectroscopy fields, researchers have explored using latent vectors for supervised tasks [24,25,26,27], employing the trained encoder as a feature extractor. However, since the process of training an autoencoder and the subsequent training of a task-specific model using latent vectors are disjoint, even if latent vectors capture features for input data reconstruction, these representations may lack features for label prediction in the subsequent task. Studies on semi-supervised autoencoders have studied to improve in performance by integrating an autoencoder with a task-specific model on the latent vector and training two models simultaneously, addressing the limitations of distinct learning phases and showing promising results.

**Figure 1:**
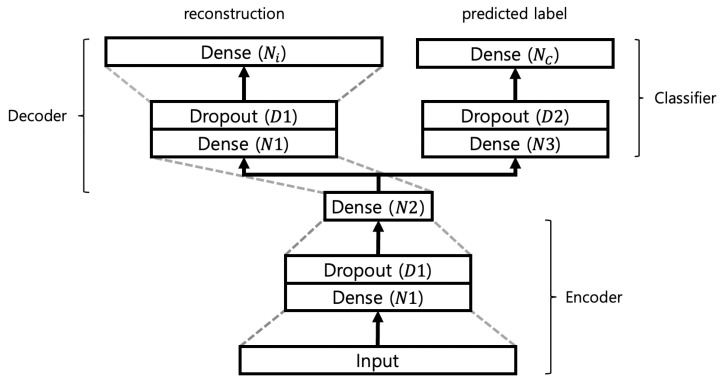
Network architecture for SSAE, which consists of encoder, decoder, and classifier.

From this perspective, we propose a deep neural network using a semi-supervised autoencoder (SSAE) to classify chemical gases based on FTIR spectra. The proposed model is a combination of an autoencoder and a classifier for gas classification. It receives the preprocessed spectrum *x*, generates the reconstruction $\hat{x}\in R^{N_{i}}$, and predicts the label $\hat{y}\in R^{N_{C}}$ indicating the type of gas. The model structure comprises an encoder, decoder, and classifier, as illustrated in Figure 1. The encoder receives the input *x* and generates a latent vector $z\in R^{N2}$ through dense layers with nodes N1 and N2. The nodes that have passed through the N2 dense layer and subsequent activation function are considered the latent vector. The decoder, receiving *z*, reconstructs $\hat{x}$ through dense layers with nodes N1 and $N_{i}$. The classifier, receiving *z*, predicts the probability $\hat{y}$ through dense layers with nodes N3 and $N_{C}$. Batch normalization and leaky ReLU are employed after all dense layers, except the N2 dense layer and output layers of the decoder and classifier. After the N2 dense layer, a sigmoid activation function is used to set the value of the latent vector between 0 and 1. The decoder output layer uses a tangent hyperbolic activation function due to the input value range between −1 and 1. Dropout is applied to prevent overfitting.

We trained the proposed model using the Adam optimizer, and the training objective was to minimize the following loss function:

$$\begin{array}{c} L\left(x,\hat{x},y,\hat{y}\right)=\frac{1}{N_{B}}\sum_{i=1}^{N_{B}}\left[\left(1-\alpha \right){∥x_{i}-{\hat{x}}_{i}∥}_{2}^{2}+\alpha \left(-\sum_{c=1}^{N_{C}}\log\left(\frac{\exp\left({\hat{y}}_{i,c}\right)}{\sum_{j=1}^{N_{C}}\exp\left({\hat{y}}_{i,j}\right)}\right)y_{i,c}\right)\right], \end{array}$$

where $N_{B}$ is the number of data points included in the batch, and $N_{C}$ is the number of gas classes to be classified. As evident in the above equation, the loss function of the proposed model is a combination of the mean squared error, representing the reconstruction error of the autoencoder, and the cross-entropy loss, representing the classification error. The model was trained to minimize both errors simultaneously. The weight of each reconstruction error and classification error was determined with α∈R.

### 2.3. Hyperparameter Optimization

The performances of deep learning models are significantly influenced by the chosen hyperparameters [40,41]. We optimized the hyperparameters through Bayesian optimization. Bayesian optimization defines a prior probability distribution for hyperparameters and an objective function representing the performance of the algorithm. Based on the objective function value for the previous parameters, the next parameters to be tried were calculated to optimize the objective function. This process was repeated for a predefined number of explorations to find the optimal hyperparameters. The Bayesian optimization method utilizes the results of previously searched parameters, enabling high-performance hyperparameter optimization compared to grid search and random search methods. A detailed description of Bayesian optimization is provided by Wu et al. [42].

The hyperparameter optimization process was accomplished through the following steps. First, hyperparameters were randomly selected, and a model using these hyperparameters was trained through 3-fold cross-validation. At this point, the data ratio for each class in each fold was set to be the same as the ratio of data for each class in the train dataset. Since our goal was to achieve high-performance gas classification, the cross-validation loss was calculated as the average cross-entropy loss of validation folds. In examining the cross-validation loss indicated by the selected hyperparameters, new parameters to be searched next were calculated within a predefined search space. In repeating this process, the hyperparameters that showed the lowest cross-validation loss among the searched hyperparameters were selected as the optimal hyperparameters.

The proposed model has seven hyperparameters: the number of neurons in each layer $\left(N1,N2,N3\right)$, dropout ratio $\left(D1,D2\right)$, weighting factor $\left(\alpha \right)$, and learning rate $\left(\eta \right)$. Autoencoders are generally structured such that the number of neurons decreases as the encoder passes through each layer, and the number of neurons increases as the decoder passes through each layer. Considering this, the search space of N1 is from 64 to 256, and that of N2 is 8 to 64. The range of N3 is the same as that of N2. The values of D1 and D2 ranged from 0 to 0.3, α ranged from 0.1 to 0.9, and η ranged from 0.01 to 0.0001 on a logarithmic scale.

## 3. Results

### 3.1. Experiment Setting

The performance of the proposed model was evaluated on the test dataset after being trained on the entire train dataset using the optimal hyperparameters selected through a hyperparameter optimization process. The number of hyperparameter combinations explored was 100, and the batch size and epochs in the training step were set to 1024 and 100 epochs, respectively. Evaluation metrics commonly used in classification performance analysis, such as the f1-score, precision, and recall, were employed. Despite introducing a hyperparameter optimization process for a detailed performance analysis of the proposed model, variations in performance may occur due to the selected optimal hyperparameters, influenced by the layer initialization value, the indexes of the folds divided in the cross-validation, and the randomness during the learning process. To enhance the general performance analysis of the model, a random state variable was set to determine the randomness in the training and hyperparameter optimization processes for reproducibility. Experiments were conducted by changing this random state five times, and the values were calculated as the average values of the evaluation metric values from the five trials.

For a comparative analysis, four conventional machine learning models (GNB, KNN, RF, SVM) and two deep learning models for classification (MLP, MLP-AE) were considered. The RF, SVM, KNN, and GNB models are commonly used for solving classification tasks using spectral data and served as performance comparison models for the deep learning models. Except for GNB, which has no hyperparameters, RF, SVM, and KNN underwent the same hyperparameter optimization process as described above. The hyperparameter search area for each model was determined by considering previous studies [43,44]. MLP and MLP-AE are deep learning models with structures similar to that of the proposed model. MLP-AE shares the same structure as the SSAE, but it first trains an autoencoder consisting of an encoder and a decoder, and then uses the latent vector generated by the trained encoder to train the classifier. As in previous studies [24,25,26,27], MLP-AE is a deep learning model that utilizes an autoencoder for feature extractor. MLP is a deep learning model for classification consisting of dense layers that incorporates an encoder and a classifier without a decoder, using leaky ReLU instead of a sigmoid as an activation function after the N2 dense layer. MLP-AE and MLP have a total of six hyperparameters that are the same as those of the SSAE, except for α, and the search spaces are set to be the same as that of the SSAE. For all comparison models, the average performance values were calculated by changing the random state in the same manner as for the SSAE.

### 3.2. Performance Analysis

Table 2 summarizes the experimental results of the seven models for all gas classes in the test dataset. The evaluation metric values presented in the table were calculated as a weighted sum, taking into account the number of data points included in each class. The results indicate that the proposed SSAE model achieved the highest performance, surpassing 0.9 in all metrics, followed by MLP and MLP-AE. Given that the test dataset comprised low-concentration spectra, achieving a performance higher than 0.9 suggests that the proposed model can deliver robust classification even in scenarios with small amounts of gas in real environments. While there have been numerous instances of using trained autoencoders as feature extractors in previous research, the experimental results here show that MLP-AE, utilizing the extracted features, exhibits the lowest performance among deep learning models. This suggests that models using a semi-supervised autoencoder, which encourages feature extraction for classification, or general deep learning classifiers consisting of dense layers can achieve superior performances compared to models using a separately trained autoencoder as a feature extractor. Among the machine learning models, KNN demonstrated the highest performance, but still exhibited a lower performance compared to the deep learning based models.

Table 3, Table 4 and Table 5 present the f1-score, precision, and recall scores of the models for each gas. The SSAE exhibited the highest f1-score for all gases except gases A and F. RF achieved the highest f1-score for gas A, with only a 0.005 difference from the SSAE, but demonstrated a lower performance than the SSAE for the other gases. Particularly noteworthy is the significantly superior performance of the SSAE compared to RF in gases B, C, and D, with the most substantial difference of 0.413 observed in gas C. Also, GNB exhibited the highest f1-score for gas F, surpassing the SSAE by 0.029, but GNB performed less effectively than the SSAE for other gases and even exhibited a score as low as 0.409 in gas C. The SSAE demonstrated high performance in most cases for precision and recall, except for in gases A, C, and F in precision and gas D in recall. However, even in cases where the SSAE did not achieve the highest performance, the difference is only marginal, up to 0.138 from the model that attained the highest performance in those instances. Consequently, the proposed SSAE consistently demonstrates the best performance, whether through analyzing the performance for all gases collectively or individually for each gas.

### 3.3. Embedding Analysis

Our model demonstrated a higher classification performance than MLP-AE and MLP. To gain a more detailed understanding of why the classification performance of the SSAE is superior, we conducted an analysis of the latent vectors, which represent the output of the encoder where the input spectrum data are embedded in low dimensions. We employed the t-SNE method to reduce latent vectors to two dimensions and visualize them, examining the distribution of the latent vectors. Additionally, we calculated a silhouette score to numerically evaluate whether the latent vectors effectively represent characteristics for classification. The silhouette score indicates how closely the data corresponding to each class are clustered together and how well the clusters of each class are separated. Within a value between −1 and 1, a score closer to 1 signifies a higher clustering performance. The visualization and silhouette score calculation using t-SNE were used to analyze both the raw spectrum and latent vectors reduced to low dimensions with MLP, MLP-AE, and the SSAE on the test dataset.

Table 6 summarizes the silhouette scores, with the scores for the deep learning models representing the average value over five iterations of changing the random state. The SSAE exhibited the highest value of 0.6083 among the raw spectra and other methods. This indicates that the latent vectors generated by the SSAE encoder form well-defined clusters among the same classes, affirming the highest classification performance in the test dataset. The raw spectrum exhibited the lowest value of 0.4053, while MLP-AE showed a higher value than the raw spectrum, confirming the efficiency of using the trained encoder as a feature extractor. However, since MLP-AE demonstrated a lower score than MLP and the SSAE, this suggests that the features expressed by the latent vector are weighted more toward expressing features for reconstruction rather than features for classification. This provides an explanation for the lower classification performance observed in MLP-AE compared to MLP and SSAE.

**Figure 2:**
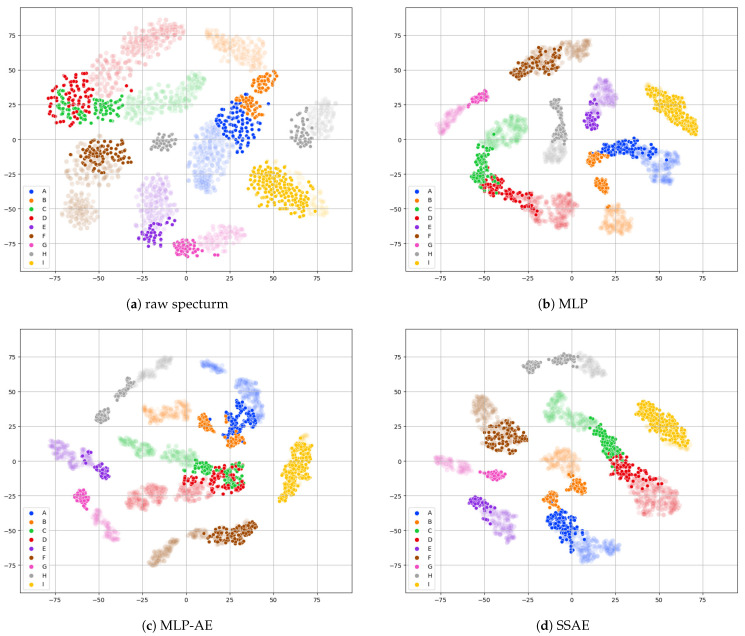
Visualization of two-dimensional reduced (**a**) raw spectrum and latent vectors generated by (**b**) MLP, (**c**) MLP-AE, and (**d**) the SSAE on the test dataset through t-SNE.

Figure 2 visually represents the raw spectrum and latent vectors of MLP, MLP-AE, and the SSAE. Opaque points denote data from the train dataset, while clear points represent data from the test dataset. In the case of raw spectrum appearing in Figure 2a, points of the same class are not densely clustered, and gases A and B appear intermixed, which is also confirmed for gases C and D. Notably, gas H exhibits significant separation between some high-concentration training dataset points and low-concentration test dataset points. Conversely, the latent vectors of the deep learning models mostly form high-density clusters for the same classes, and the training and test data points are closely located. However, for MLP-AE, it can be observed that the test data points for gases C and D are partly mixed, whereas MLP and the SSAE demonstrate relatively clear distinctions between those points. This discrepancy is attributed to the fact that the autoencoder of MLP-AE is trained without labels for gas classification, visually illustrating why its classification performance is lower than those of MLP and the SSAE. These analyses provide evidence supporting the claim that the proposed SSAE model outperforms other models, as revealed by the visualization of latent vectors and silhouette clustering scores.

## 4. Conclusions

In this paper, we introduced the SSAE model for classifying gases present in FTIR spectra. The SSAE utilizes a semi-supervised autoencoder framework structure, jointly training an autoencoder for unsupervised input reconstruction and a classifier for supervised gas classification. In this combined framework, the autoencoder is trained not only to capture the semantics of the input data but also to represent features crucial for classification. The proposed model was trained on high-concentration gas spectra obtained in the laboratory, and its classification performance was evaluated on low-concentration gas spectra. In a statistical experimental environment, the SSAE demonstrated a superior classification performance compared to those of classical machine learning models and deep learning based classification models. The factors that enabled the SSAE to achieve the highest classification performance were confirmed by analyzing the distribution of latent vectors embedding the input data. The SSAE exhibits the ability to generate denser cluster distributions in latent vectors compared to deep learning classifiers that utilize a learned autoencoder or are composed of dense layers. This capability contributes to its high performance in classification, a conclusion supported by silhouette scores indicating the clustering performance and visualizations of the latent vectors.

## Figures and Tables

**Table 1:** Injected amounts and the number of samples for each gas in the training and test datasets.

Chemical Gas	Train Dataset		Test Dataset	
	Amounts (µL)	Samples	Amounts (µL)	Samples
A	6, 8, 10, 15	400	2, 4	200
B	6, 8, 10	300	2, 4	200
C	6, 8, 12, 15	400	2, 4	200
D	6, 8, 12, 15, 20	500	2, 4	200
E	60, 90, 120	300	30	100
F	60, 80, 120, 160	400	20, 40	200
G	40, 60	200	20	100
H	12, 16	200	4, 8	200
I	20	100	2, 4, 6, 8	400
total		2800		1800

**Table 2:** Performance evaluation scores of models on test dataset.

Model	f1-Score	Precision	Recall
GNB	0.7705	0.8705	0.7844
KNN	0.8532	0.8894	0.8584
SVM	0.7404	0.8134	0.7718
RF	0.7809	0.8774	0.7875
MLP	0.8902	0.9187	0.8938
MLP-AE	0.8709	0.9095	0.8758
SSAE	0.9116	0.9313	0.9133

**Table 3:** F1-score of models for each gas on test dataset.

Model	A	B	C	D	E	F	G	H	I
GNB	0.8232	0.8130	0.4790	0.6546	0.7490	0.8908	0.5294	0.6348	1.0000
KNN	0.8190	0.7104	0.7735	0.8041	1.0000	0.8323	0.9969	0.7412	1.0000
SVM	0.8094	0.5133	0.3617	0.6948	0.9812	0.7400	0.7929	0.6570	1.0000
RF	0.8918	0.5936	0.4739	0.6186	0.9658	0.7323	0.9869	0.7419	1.0000
MLP	0.8695	0.8014	0.8537	0.8857	0.9990	0.8415	1.0000	0.7681	0.9960
MLP-AE	0.8275	0.7343	0.7750	0.8473	0.9932	0.8589	0.9927	0.8026	1.0000
SSAE	0.8868	0.8437	0.8888	0.9089	1.0000	0.8610	1.0000	0.8152	1.0000

**Table 4:** Precision of models for each gas on test dataset.

Model	A	B	C	D	E	F	G	H	I
GNB	0.7449	1.0000	1.0000	0.4877	0.5988	0.8032	1.0000	1.0000	1.0000
KNN	0.6950	1.0000	0.8553	0.7416	1.0000	0.7130	1.000	1.0000	1.0000
SVM	0.6927	0.8000	0.8000	0.5442	0.9786	0.5948	0.8000	1.0000	1.0000
RF	0.9359	0.9634	0.9774	0.4645	0.9718	0.5780	0.9839	1.0000	1.0000
MLP	0.7728	0.9988	0.9843	0.8028	0.9980	0.7265	1.0000	1.0000	0.9923
MLP-AE	0.7062	1.0000	0.9912	0.7418	0.9869	0.7528	1.000	1.0000	1.0000
SSAE	0.7978	1.0000	0.9794	0.8481	1.0000	0.7564	1.0000	1.0000	1.0000

**Table 5:** Recall of models for each gas on test dataset.

Model	A	B	C	D	E	F	G	H	I
GNB	0.9200	0.6850	0.3150	0.9950	1.0000	1.0000	0.3600	0.4650	1.0000
KNN	0.9980	0.5539	0.7080	0.8800	1.0000	1.0000	0.9940	0.5890	1.0000
SVM	0.9740	0.3780	0.2350	0.9700	0.9840	1.0000	0.7860	0.5049	1.0000
RF	0.8520	0.4309	0.3130	0.9269	0.9600	1.0000	0.9900	0.5900	1.0000
MLP	0.9990	0.6799	0.7539	0.9880	1.0000	1.0000	1.0000	0.6240	1.0000
MLP-AE	1.0000	0.5820	0.6440	0.9930	1.0000	1.0000	0.9860	0.6710	1.0000
SSAE	1.0000	0.7320	0.8170	0.9820	1.0000	1.0000	1.0000	0.6890	1.0000

**Table 6:** The silhouette scores for the raw spectrum and latent vectors generated by MLP, MLP-AE, and the SSAE on the test datatset.

	Raw Spectrum	MLP	MLP-AE	SSAE
silhouette score	0.4053	0.5590	0.4653	0.6083

## Data Availability

The data presented in this study are available upon request from the corresponding author. The data are not publicly available due to legal restrictions.

## Abbreviations

The following abbreviations are used in this manuscript:

GNB	Gaussian naive Bayes;
KNN	K-nearest neighbor;
SVM	Support vector machine;
RF	Random forest;
MLP	Multi-layer perceptron;
MLP-AE	Multi-layer perceptron with trained autoencoder;
SSAE	Semi-supervised autoencoder.
